# Supplementary figures and images for: ­­­­­­Widespread conservation and lineage-specific diversification of genome-wide DNA methylation patterns across arthropods
Source: PLoS Genet. 2020 Jun 25;16(6):e1008864. doi: 10.1371/journal.pgen.1008864 (PMC7343188; doi:10.1371/journal.pgen.1008864)

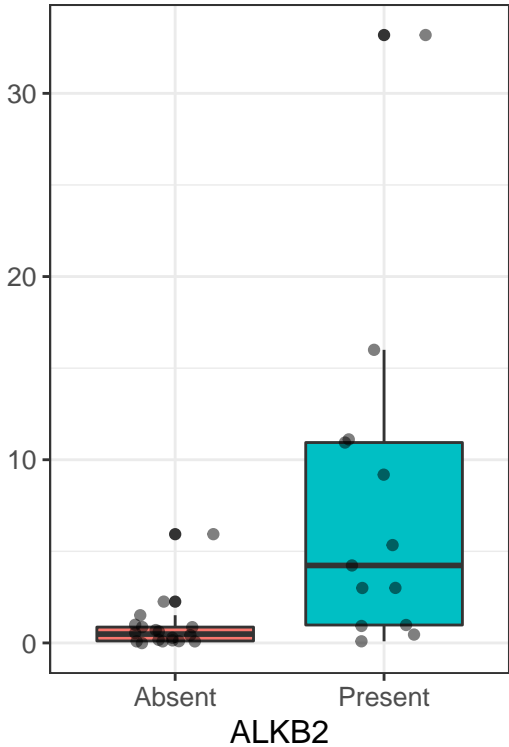

Supplement: S1 Fig — Boxplot showing genome-wide methylation levels in 31 arthropod species with and without ALKB2. (PDF) [file pgen.1008864.s001.pdf]

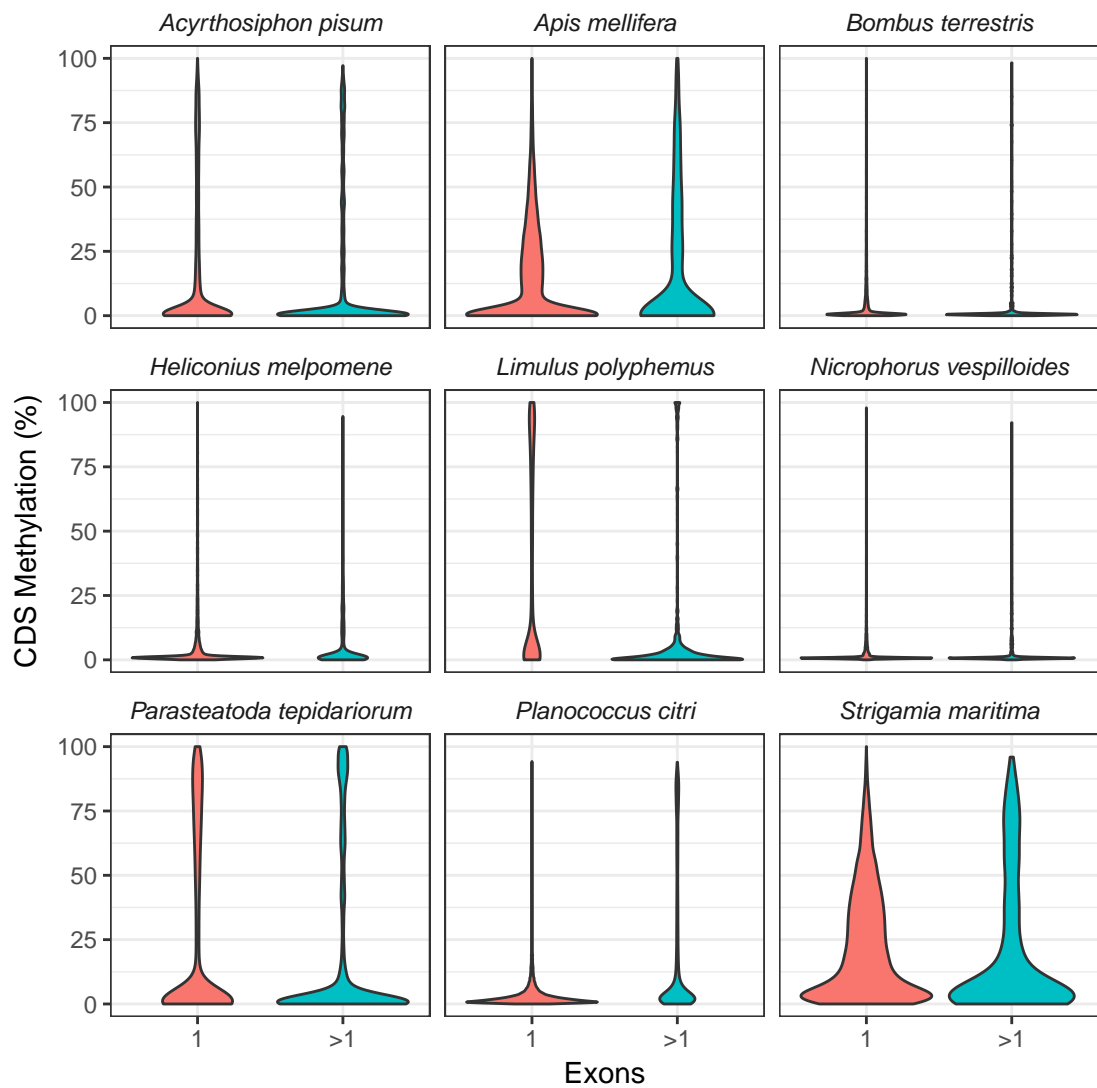

Supplement: S5 Fig — (PDF) [file pgen.1008864.s005.pdf]

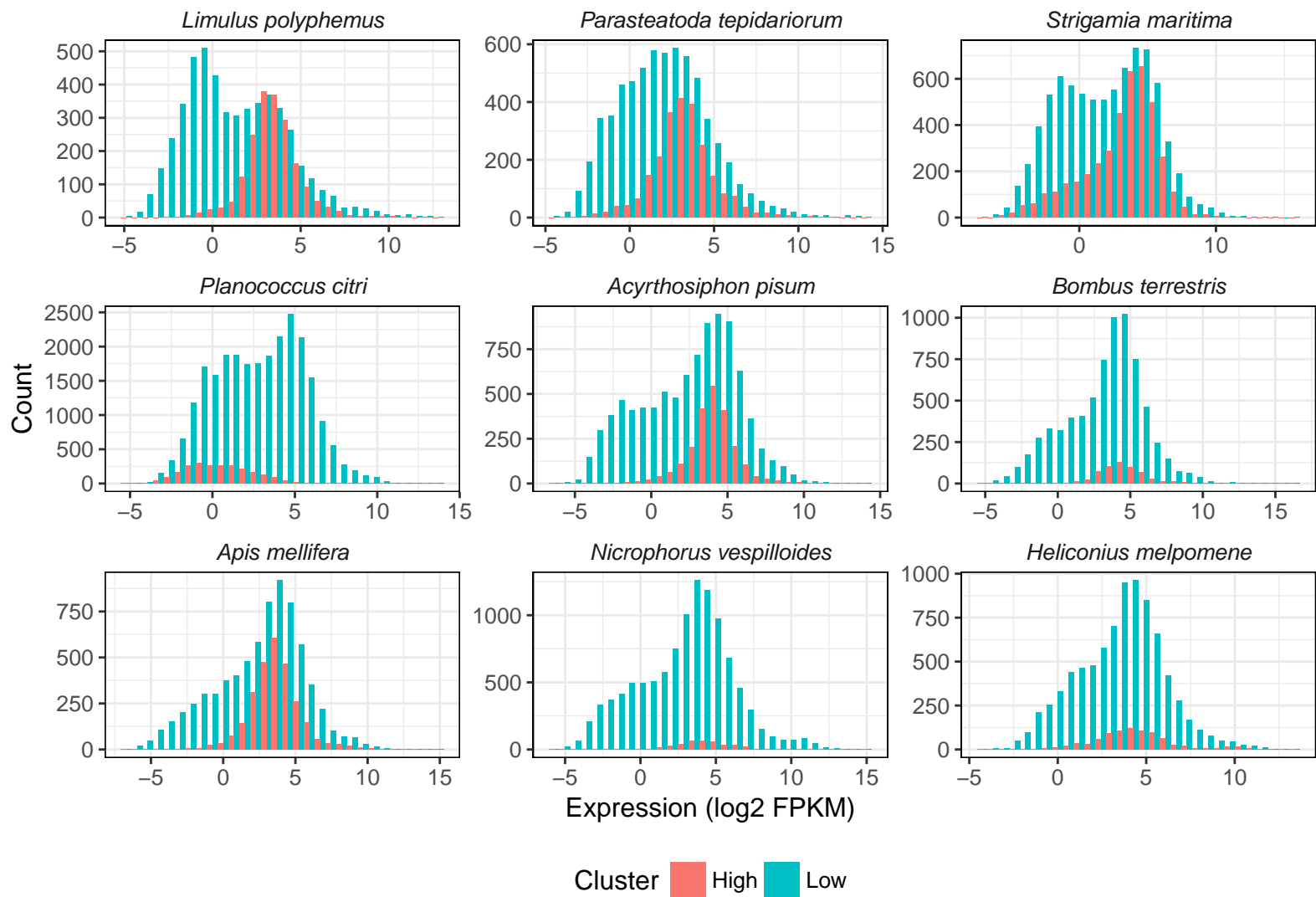

Supplement: S6 Fig — (PDF) [file pgen.1008864.s006.pdf]

**A**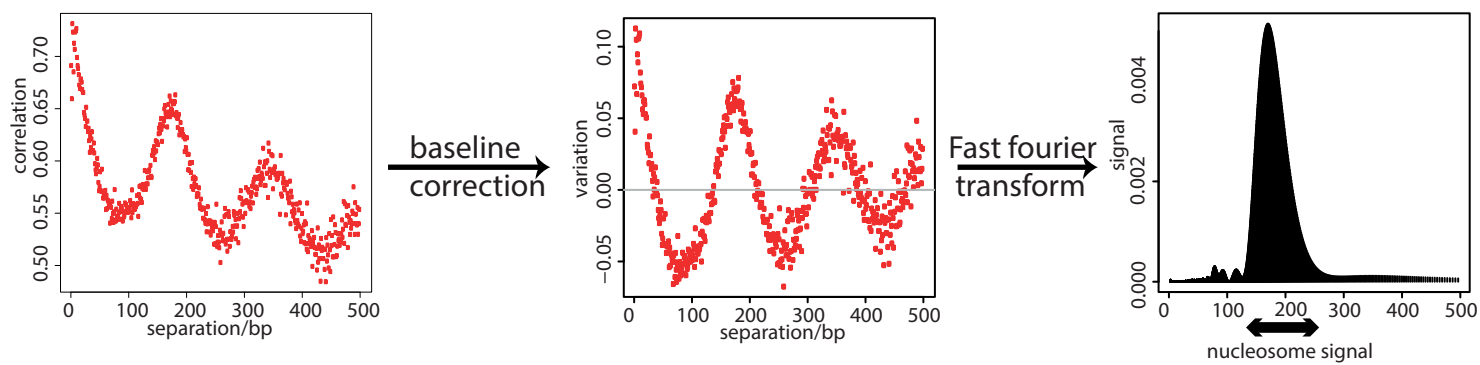**B**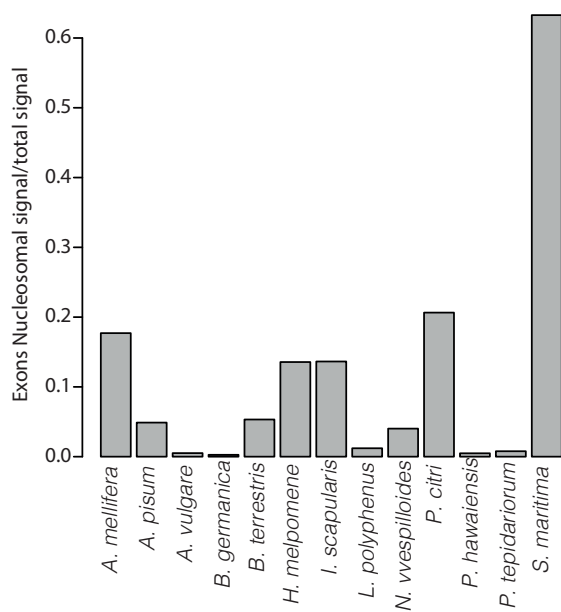**C**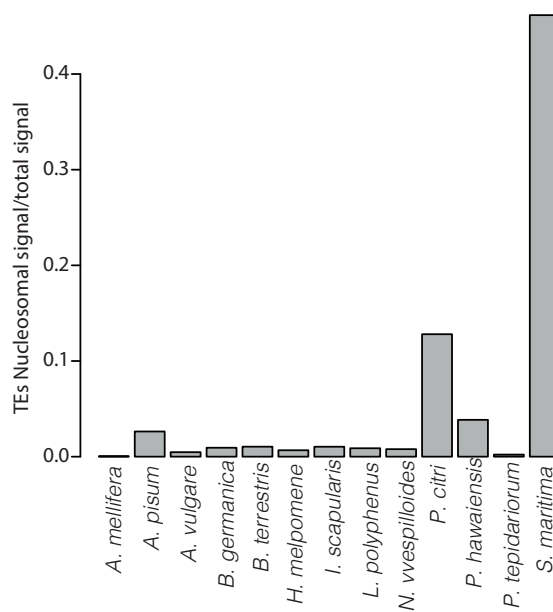

Supplement: S7 Fig — A) Diagram of method to convert from the pattern into the magnitude of the nucleosome periodicity B, C) Periodicity in coding sequences and transposable elements respectively. (PDF) [file pgen.1008864.s007.pdf]

**A****exon:1**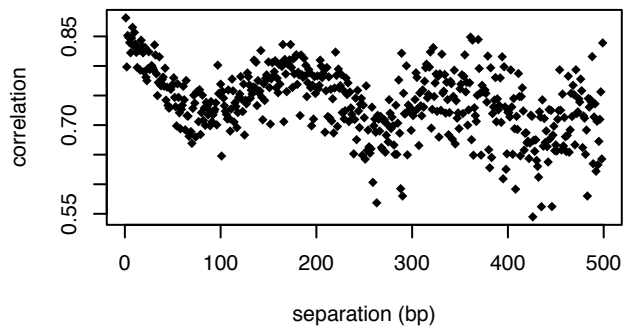**exon:2**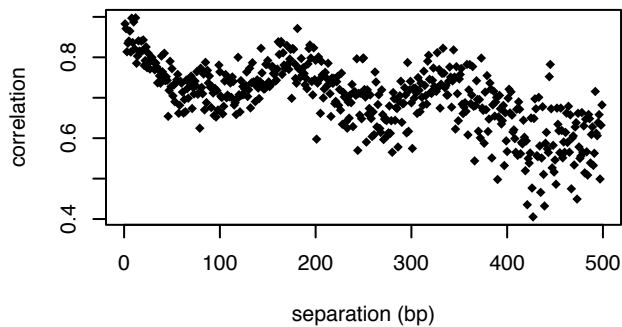**exon:3**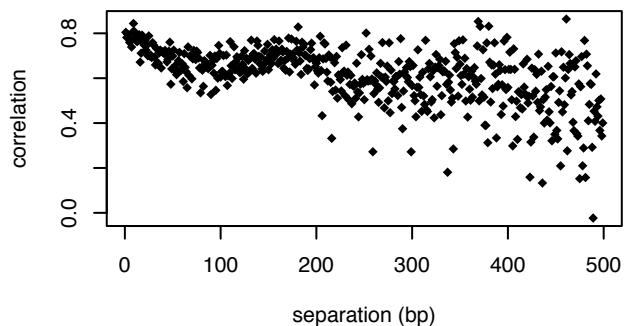**exon:4**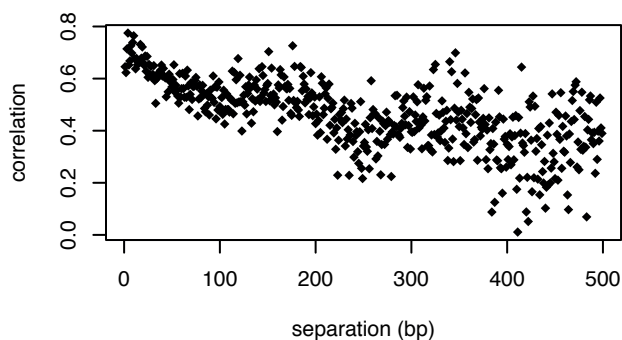**B****intron:1**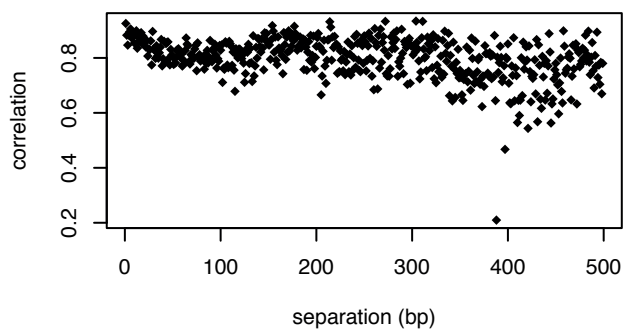**intron:2**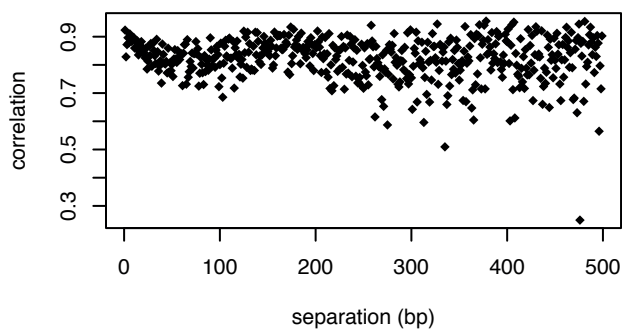**intron:3**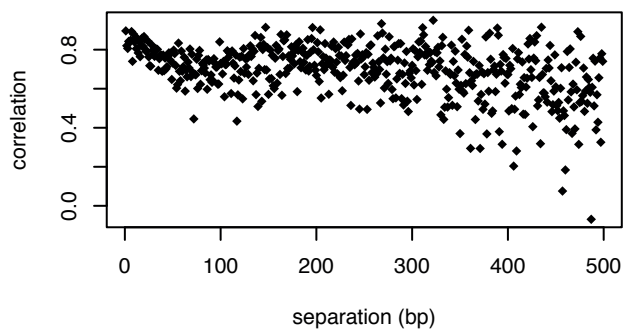**intron:4**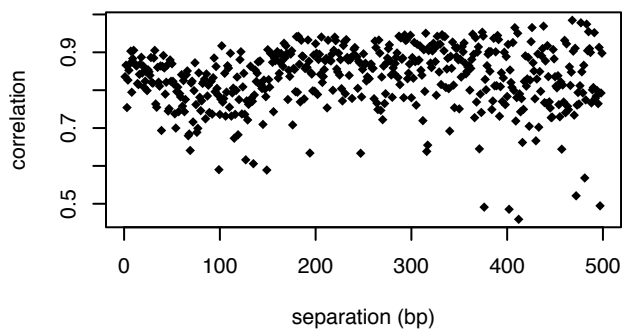

Supplement: S8 Fig — S. maritima exons 1 to 4 (A) and introns 1 to 4 (B) are shown for comparison. (PDF) [file pgen.1008864.s008.pdf]

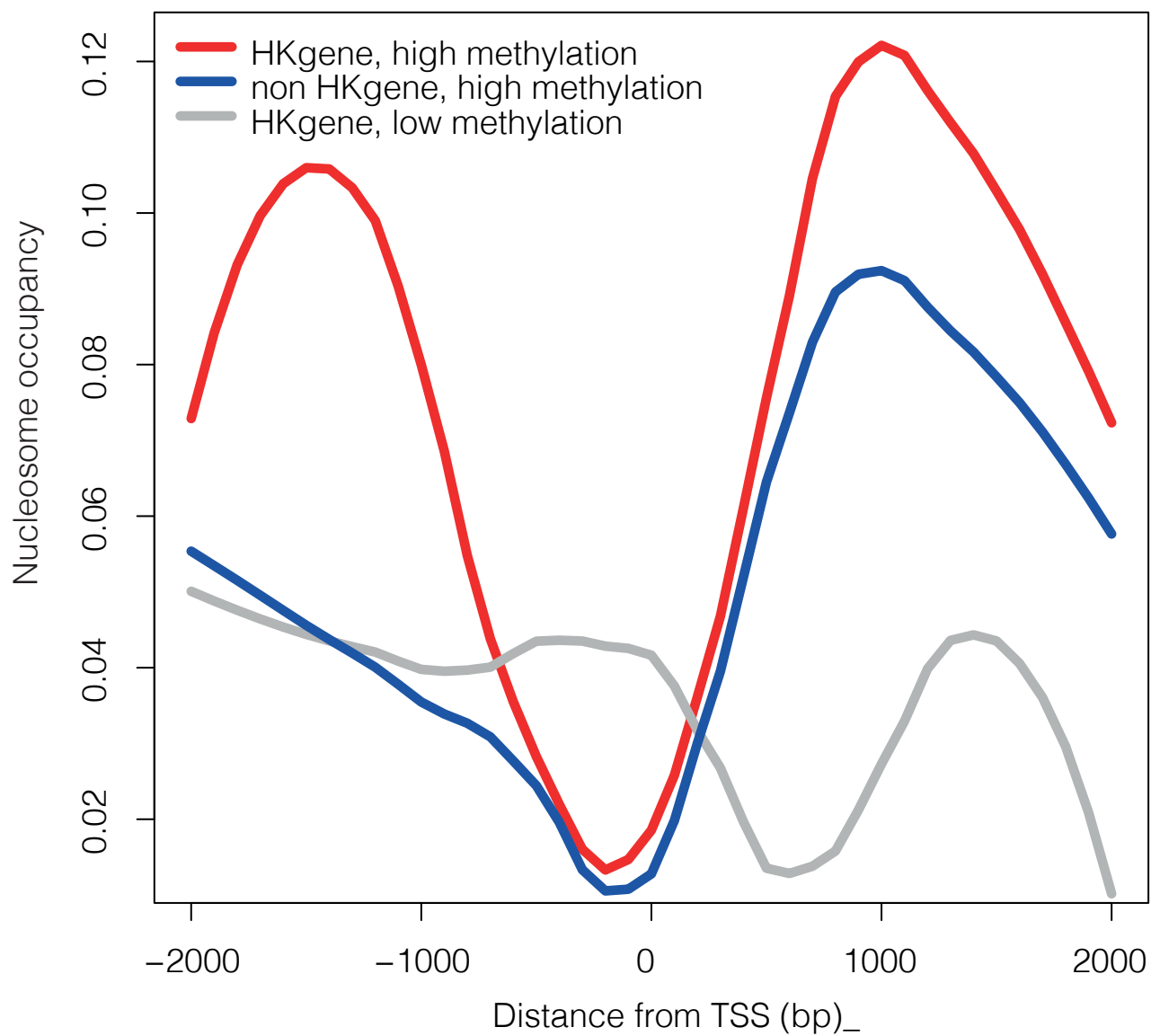

Supplement: S9 Fig — Both housekeeping genes and non-housekeeping genes that are methylated genes show enhanced nucleosome occupancy at the +1 nucleosome. (PDF) [file pgen.1008864.s009.pdf]
