# Supplementary material for: ­­­­­­Widespread conservation and lineage-specific diversification of genome-wide DNA methylation patterns across arthropods
Source: PLoS Genet. 2020 Jun 25;16(6):e1008864. doi: 10.1371/journal.pgen.1008864 (PMC7343188; doi:10.1371/journal.pgen.1008864)
Supplement: S2 Fig — TEs are shown in pink, flanking sequence in white. (PDF) [file pgen.1008864.s002.pdf]

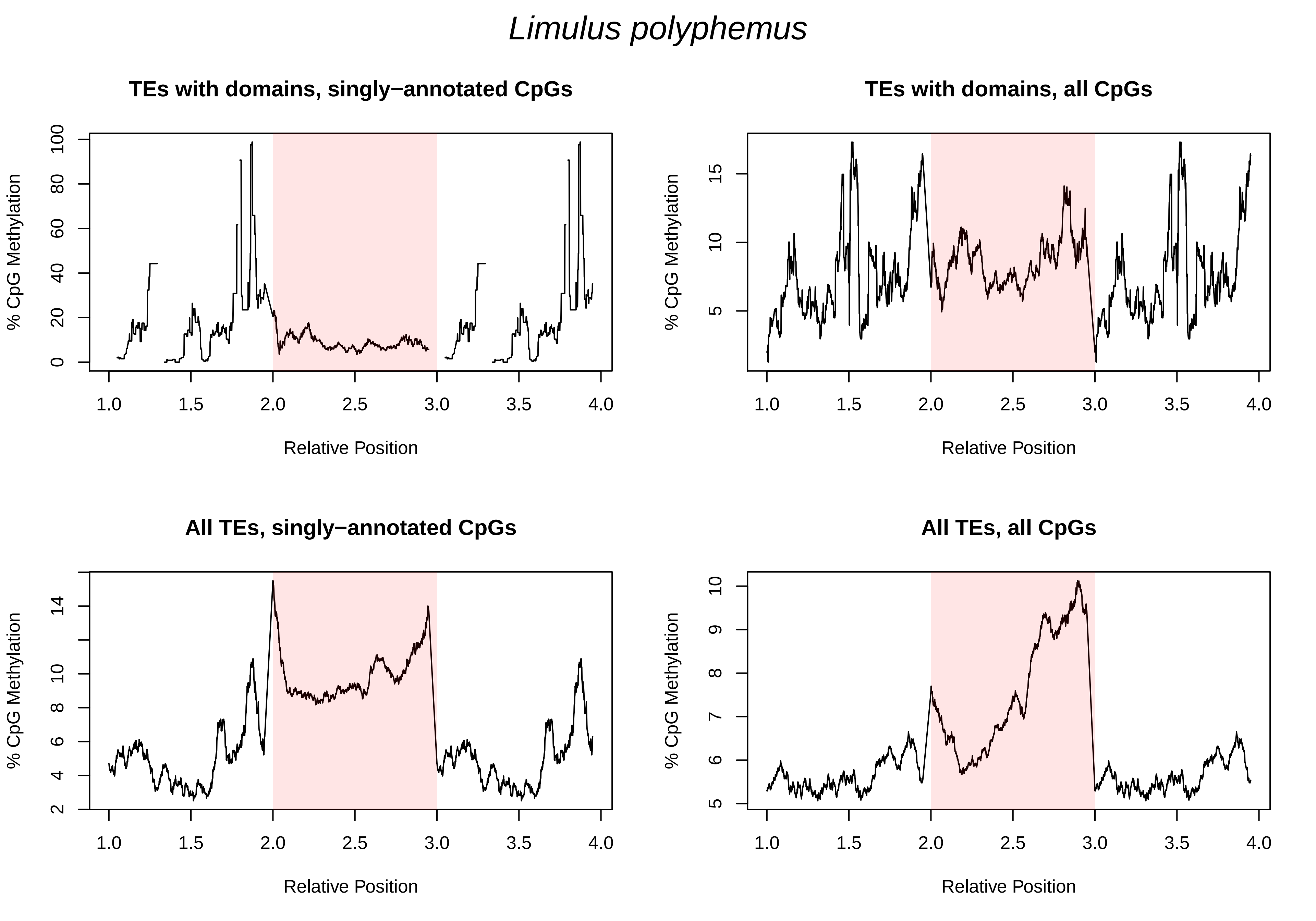
Limulus polyphemus
TEs with domains, singly−annotated CpGs
TEs with domains, all CpGs
All TEs, singly−annotated CpGs
All TEs, all CpGs
% CpG Methylation
Relative Position
0
20
40
60
80
100
5
10
15
2
4
6
8
10
14
5
6
7
8
9
10
1.0
1.5
2.0
2.5
3.0
3.5
4.0

Parasteatoda tepidariorum

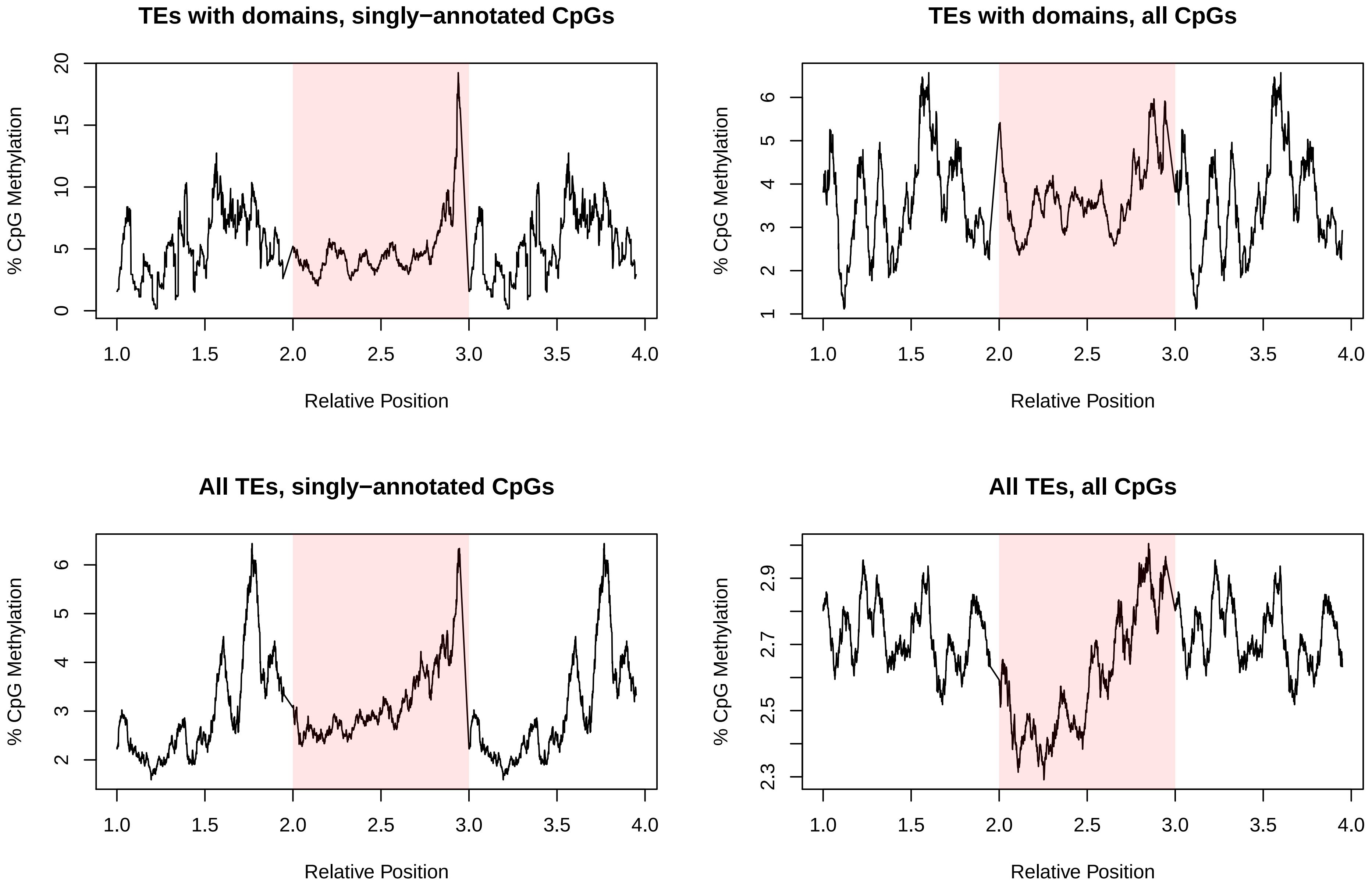

Ixodes scapularis

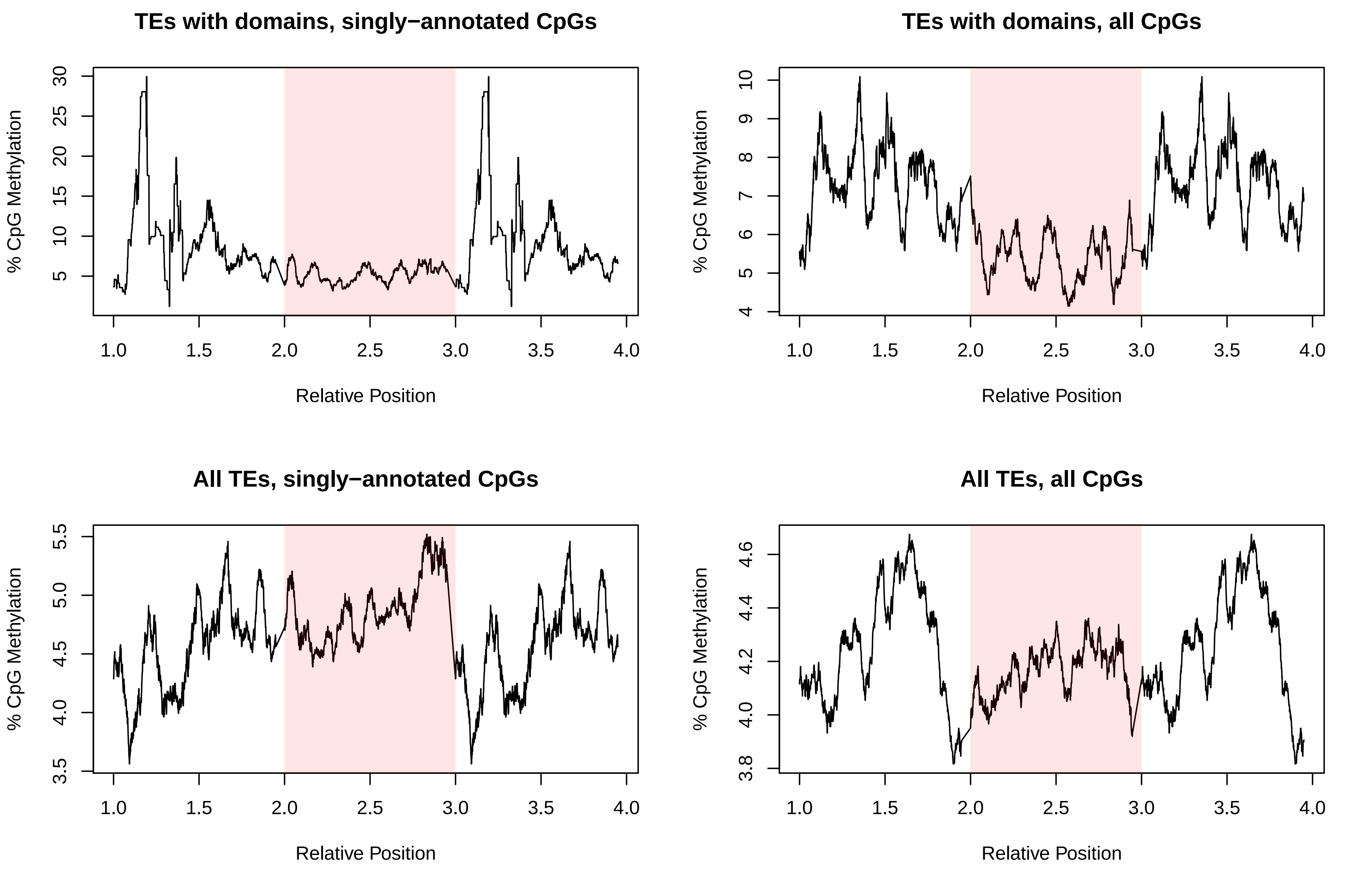

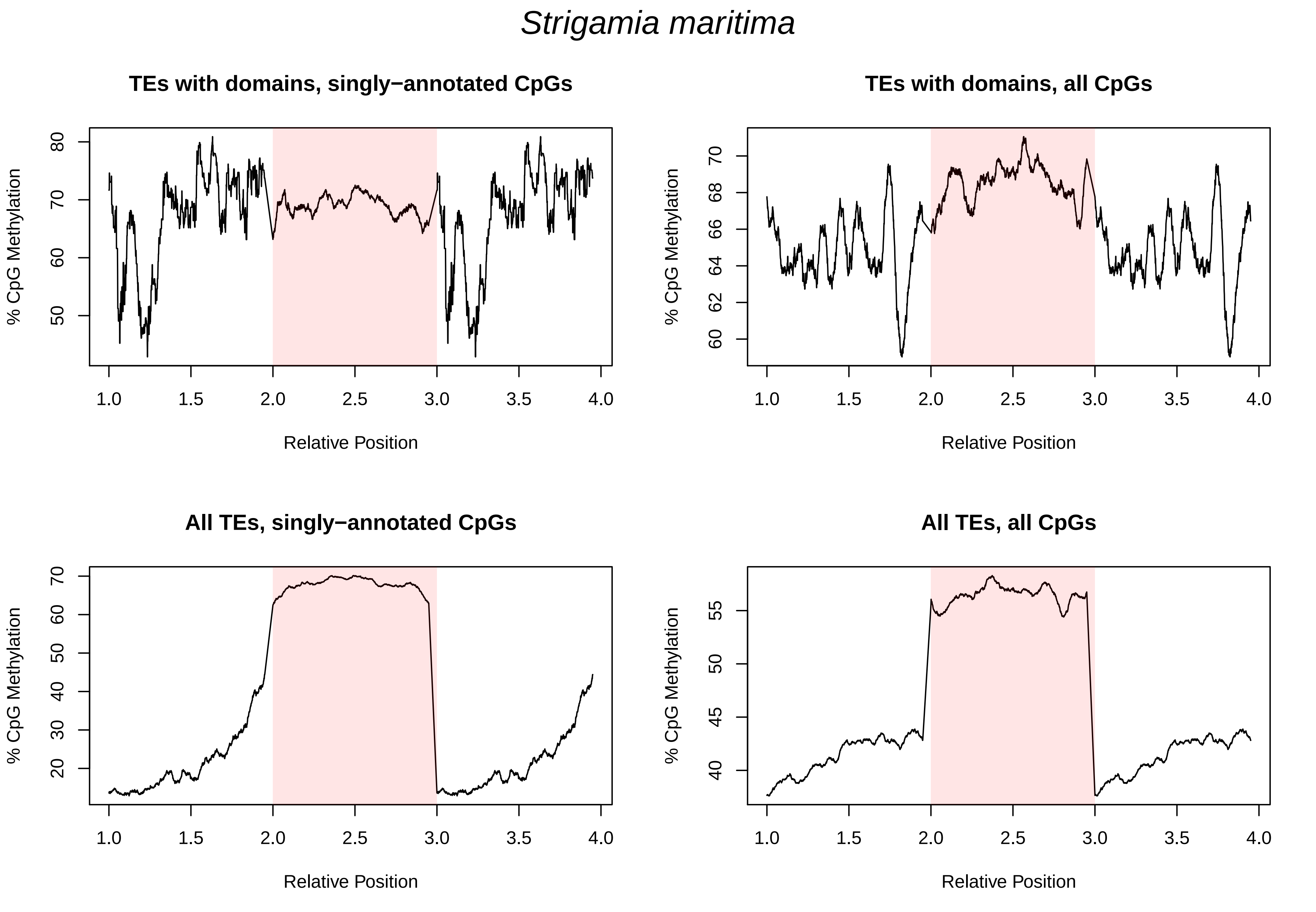
Strigamia maritima
TEs with domains, singly−annotated CpGs
TEs with domains, all CpGs
All TEs, singly−annotated CpGs
All TEs, all CpGs
% CpG Methylation
Relative Position
1.0
1.5
2.0
2.5
3.0
3.5
4.0

Blattella germanica

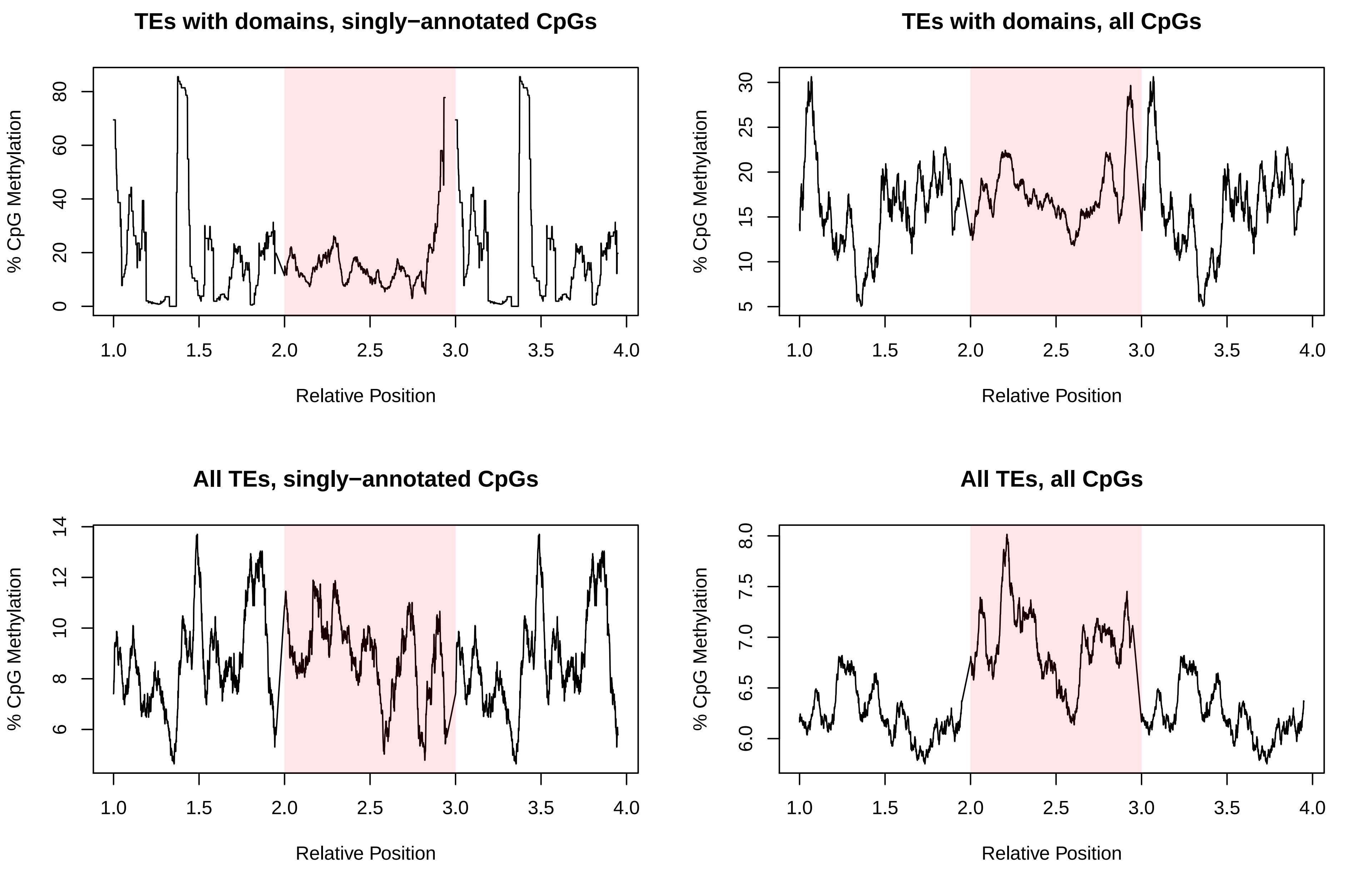

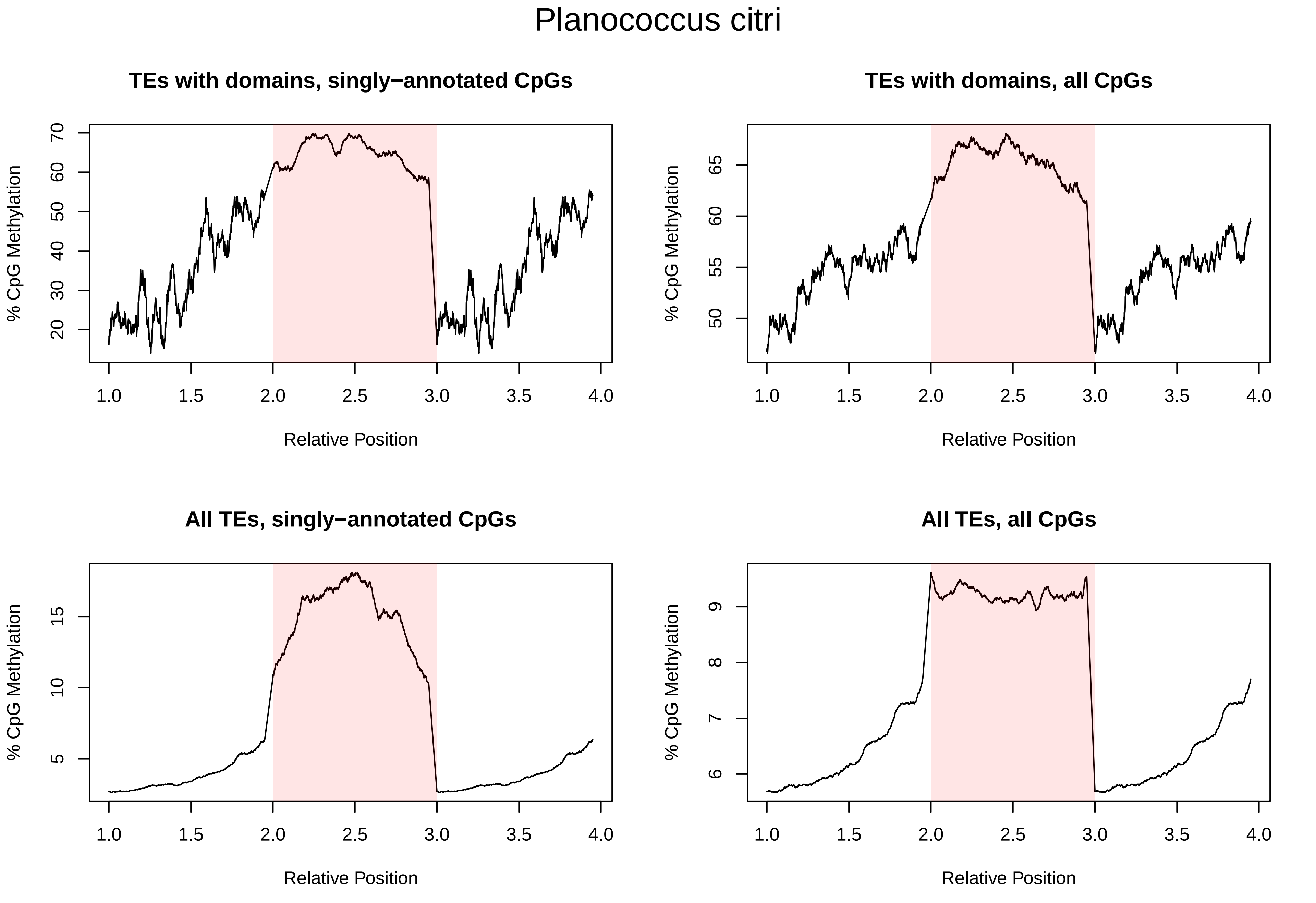
Planococcus citri
TEs with domains, singly−annotated CpGs
TEs with domains, all CpGs
All TEs, singly−annotated CpGs
All TEs, all CpGs
% CpG Methylation
Relative Position
1.0
1.5
2.0
2.5
3.0
3.5
4.0
20
30
40
50
60
70
50
55
60
65
5
10
15
6
7
8
9
