# Supplementary material for: ­­­­­­Widespread conservation and lineage-specific diversification of genome-wide DNA methylation patterns across arthropods
Source: PLoS Genet. 2020 Jun 25;16(6):e1008864. doi: 10.1371/journal.pgen.1008864 (PMC7343188; doi:10.1371/journal.pgen.1008864)
Supplement: Attachment — Submitted filename: Reviewerresponses_ver2.docx [file pgen.1008864.s011.docx]

Reviewer #1: The study be Lewis and colleagues’ profiles genome-wide DNA methylation at single base resolution in species distributed broadly across the arthropod phylum. The observed repeated loss of DNA methylation and its associated DNMTs in some species indicates that the most recent common ancestor possessed DNA methylation. There is widespread evidence that DNMT3 is frequently lost across this phylum, yet, DNMT1 is sufficient to maintain the presence of CpG methylation in the genome. In a couple of species they observed independent acquisition of promoter methylation which was associated with TE acquiring. This is a very exciting observation. Curiously, DNA methylation in arthropods is highly enriched in gene bodies, especially within exons. The authors observed a periodicity associated with DNA methylation that very likely reflects the distribution of nucleosomes. To test this possibility, the authors took advantage of the fact that genes with methylation in arthropods are generally conserved. This provided an opportunity to use nucleosome positioning data from Drosophila, a species without DNA methylation. The authors found that nucleosomes were much better positioned in genes that are generally gene body methylated across the arthropod phylum.

Overall, this is a very nice and timely study that contributes to our growing knowledge that although the DNA methylation machinery is generally conserved, the manner in which the genome uses it are quite diverse. I find it very exciting that the authors found evidence for independent acquisition of promoter methylation that can influence gene expression. These unique species will provide clues on how this process has evolved. The minor comments below are intended to improve upon this nice contribution to the field.

1. I could not find any summary sequencing statistics associated with the WGBS data. Number of aligned reads, coverage, sodium bisulfite conversion efficiencies, etc.

**>>We have added a table with this data in to the manuscript (Supplemental Table 1)**

2. Although I really like the use of the comparative analysis using Drosophila nucleosome positioning data, I think the manuscript should be carefully modified to indicate that there is not yet direct data of nucleosome positioning in a species that possesses DNA methylation (at least from this study). I believe the results will be the same, but until the experiment is done the language should be carefully crafted to make this clear.

**>>We thank the reviewer for this point- we have clarified this point in the revised version of the text both in the results and in the discussion:**

“We did not have genome-wide nucleosome positioning data for the majority of species so decided to investigate high-quality nucleosome positioning from *Drosophila*”

**and**

“To test these ideas directly will require detailed examination of nucleosome positioning data across arthropods alongside knockouts of DNA methyltransferases”

3. In the species that independently acquire DNA methylation do the DNA methyltransferase acquire additional domains that could influence its ability to target different regions of the genome? Please add this to the discussion.

**>>We thank the reviewer for this suggestion. In the revised version of the manuscript we identified some potentially interesting differences in the domains contained in DNMT1. In particular, both S. maritima and P. citri, which have gained TE methylation, possess at least one DNMT1 homologue that lacks the CXXC Zinc finger region which has previously been shown to be important in restricting DNMT1 activity to hemimethylated DNA. It is tempting to speculate that loss of this domain independently in the two species might be associated with methylation of TEs, although further examples would be needed to confirm this. Discussion of this point is now included in Figure 2-Supplement 2 and 3 and on page 7, lines 7:22.**

4. I would also consider that in addition to targeting methylation to exons an alternative explanation that DNA methylation maintenance might be more efficient at these regions. Targeting and maintenance are a bit different and these possibilities could be discussing.

**>>This is a fair point- we can’t untangle these two possibilities and have included reference to this in the discussion:**

“Importantly however, it should be noted that an alternative explanation for different patterns of methylation is differences in the efficiency of maintaining methylation through cell division, which may vary across the genome due to differences in the targeting of DNMT1. Future work using specific knockouts of DNMT1 or DNMT3 in arthropod species would be one way to distinguish between these explanations.”

5. In the first figure many methylomes are of low coverage, but a high coverage methylation that was not included is from Oncopeltus fasciatus.
**We thank the reviewer for this suggestion. O. fasciatus has now been added to figure 1. Additionally, we have added the crayfish P. virginalis to figure 1 (see Reviewer 2 below). Both species have also now been included in the analysis of how the presence or absence of ALKB2 relates to DNA methylation levels (Supplemental Figure 1).**

Reviewer #2: Sarkies et al. analyze DNA methylation patterns in a range of arthropod species. They find that most arthropods have low levels of TE methylation, as is common in invertebrates, but that two species have preferential TE methylation that likely evolved independently. This is especially convincing in S. maritima. The authors also provide evidence that promoter methylation causes transcriptional silencing in these species and suggest that this is linked to methylation as a mechanism of TE silencing. A recent paper from Ryan Lister’s lab argues this for a sponge (de Mendoza et al, Nat Ecol Evol. 2019) and echoes a much earlier proposal for the evolution of TE silencing by methylation in vertebrates (Zemach et al., Science 2010). Still, it’s valuable to have additional examples that demonstrate the ease with which this evolutionary development can occur in animals.
**>>We thank the reviewer for their positive response to this important aspect of our manuscript.**
The authors’ treatment of gene body methylation is of more concern. The association of gene body methylation with constitutively expressed, evolutionarily conserved housekeeping genes and its corresponding depletion from variably expressed genes is probably the best-known gene body methylation characteristic in plants and animals. The initial description of this phenomenon was published by Steve Jacobsen’s lab in Arabidopsis (Zhang et al., Cell 2006); more elaborate characterizations in Arabidopsis and honeybee were published a little later (Aceituno et al., 2008; Elango et al., 2009). Many more papers have been published since, as illustrated by this quote from a recent paper that reported this phenomenon in the coral Acropora millepora (Dixon et al., Mol Biol Evol 2016):

“We showed that strongly methylated genes in A. millepora tend to have constitutive and ubiquitous functions and are less likely to be differentially expressed across developmental stages and environmental regimes. These results corroborate earlier findings from diverse taxa including plants (Aceituno et al. 2008; Coleman-Derr and Zilberman 2012; Takuno and Gaut 2012), cnidarians (Sarda et al. 2012; Dimond and Roberts 2016), mollusk (Gavery and Roberts 2010, 2013), arthropods (Elango et al. 2009; Wang et al. 2013), and a basal chordate (Suzuki et al. 2013; Keller et al. 2015). The relationship with differential expression in response to environmental regimes suggests the intriguing possibility that gbM could modulate gene expression plasticity.”

Since then, this phenomenon has been reported in several more papers (probably not a complete list): Kvist et al., Genome Biol Evol 2018 for Daphnia; Gatzmann et al., Epigenetics & Chromatin 2018 for crayfish; Liu et al., Genes 2019 for spiders; Harris et al., Epigenetics & Chromatin 2019 for honeybee. Note that all these papers cover arthropods (and half are not cited). The crayfish paper in particular may complicate the authors’ conclusions about crustaceans.

Therefore, it is not “remarkabl[e]” that “the same set of genes are likely to be methylated in all species” or that “these genes have characteristic patterns of expression”. This has been extensively demonstrated in many plant and animal (including arthropod) species. The authors are not confirming an “earlier speculation” as they put it in the discussion, but restating well-supported conclusions from earlier work.
**>>We apologise that we did not cite all the studies showing evidence for housekeeping gene methylation and we have included many more references to previous publications in the revised version of the manuscript. We have also added two further species, P. virginalis and O. fasciatus, to the analysis of genome-wide methylation across arthropods in Figure 1. Nevertheless, the reviewer will agree with us that it is remarkable that the same set of genes is selected for methylation in very distantly related species- we did not imply by this phrase that it was unexpected before our study, simply that it is worth taking note of and requires a special explanation. We have removed the word ‘remarkably’ from the abstract. Importantly, several aspects of our work here are novel.**

1) **Other studies have identified housekeeping genes as being methylated in animal genomes but to our knowledge this is the first study that shows there is a tendency for *the same set of housekeeping genes* to be more likely to be methylated across arthropods, stretching back to very distantly related members of the phylum, as we do in Figure 5.**

**2) we link for the first time the tendency for housekeeping genes to have distinct nucleosome positioning to their propensity to become methylated (we have done new analyses to support this - see below).**

**3) We link this, for the first time, to differences in the initiation of transcription- broad initiation zones at methylated genes and focussed initiation at housekeeping genes.**

**In summary, we did not intend to claim that our observation of housekeeping genes being more likely to be methylated was novel and we apologise if we inadvertently gave this impression. Nevertheless, we do make some important extensions to this observation, which we believe provide some new avenues for understanding the reasons why housekeeping genes are selected.**

There are similar issues about the association between genic DNA methylation and nucleosomes. First, the authors’ claim that “nucleosome positioning influences DNA methylation levels across arthropods” (line 3, page 10) is far stronger than the data justify. Instead, the authors report a correlation with nucleosome positioning in a species (Drosophila) that lacks DNA methylation. They can’t distinguish if nucleosome positioning influences methylation, vice versa, or both. Second, the relationship between methylation and nucleosomes is hardly unexplored. Of most relevance, enrichment of methylation over nucleosomes and the corresponding nucleosomal periodicity of methylation have been reported long ago for Arabidopsis and humans (Chodavarapu et al., Nature 2010). An enrichment of gene body methylation at nucleosomes has been described more recently in Arabidopsis (Lyons and Zilberman, eLife 2017). Preferential methylation of nucleosomes in genes is not a new finding. Other strong associations between nucleosomes and DNA methylation (cytosine and adenine) have also been reported, including the ability of methylation to influence nucleosomes (for example, Huff and Zilberman, Cell 2014; Fu et al., Cell 2015; Beh et al., Cell 2019). Finally, the authors are creating the appearance of novelty by associating known features of housekeeping gene transcription and nucleosome organization with a known feature of gene body methylation (enrichment in constitutively expressed housekeeping genes). Because gene body methylation is concentrated in housekeeping genes, it will of necessity correlate with features of these genes.

**>>We do appreciate that there is prior evidence of a connection between nucleosome positioning and DNA methylation- indeed we made this point ourselves. However, our study adds important new information here.**

1) **We are documenting a long-range periodicity of ~160bp which has not been explicitly observed genome-wide in other animals (though one can detect it in a subset of methylated regions in mammalian genomes, as cited in the submitted version).**
2) **It is not at all obvious that the association between nucleosomes and methylation patterns seen in mammals and plants should be conserved in arthropods, especially as methylation is generally much lower. Reporting this is therefore in our view an important observation.**

**3) our study makes a potential link between nucleosomes, DNA methylation and the propensity of housekeeping genes to be methylated. Importantly, we show that nucleosome and transcriptional initiation characteristics of methylated genes are conserved in the same genes in Drosophila, which doesn’t have DNA methylation. This argues strongly that these factors act upstream of methylation.**

**4) In the revised version of the manuscript we have added further analyses that addresses the question of causality between methylation and nucleosome positioning. We use a set of housekeeping genes defined from Drosophila. As expected, methylated genes overlap with housekeeping genes highly significantly. However, this allows us to specifically examine methylated genes that are not housekeeping genes. Crucially, this set of genes shows nucleosome positioning patterns that are very similar to methylated genes that are housekeeping genes. This strongly suggests that nucleosome positioning is associated with DNA methylation and, separately, is associated with the propensity for genes to have housekeeping functions. Thus our work prompts a new angle with which to consider the relationship between housekeeping genes and DNA methylation propensity within arthropods, which may also have relevance to methylation of genes outside of arthropods.**

This paper contains interesting observations about the evolution of DNA methylation and could be a valuable addition to the field if the authors were to place their finding properly within the existing literature. However, given the relatively low novelty of the conclusions, I feel this paper is much more suitable for a journal like Mol Biol Evol or Epigenetics & Chromatin (in which many of the relevant recent papers have been published).

**>>We thank the reviewer for this response. In the revised version we have cited many more references, especially from the plant literature, which have bearing on our work to ensure that the context of our work is more clearly stated. Nevertheless, we would argue that the generality of our observations, in combination with the novel aspects of our work that we highlight above, make it suitable for the remit of PLoS Genetics.**

Reviewer #3: DNA methylation (5MeC) is an epigenetic regulatory mechanism, which among eukaryotes displays great variability in terms of genomic content and function. The majority of studies focusing on 5MeC to date have been carried out on vertebrate model organisms that exhibit hypermethylated genomes. Due to the recent increase in accessibility of genomic sequencing technologies, more invertebrate organisms have had their genomes and DNA methylomes sequenced. While this provided important insights into the evolutionary conservation and divergence of 5MeC, many open questions still remain. In the current manuscript Lewis et al generate base-resolution DNA methylome maps of the highly diverse Arthropod phylum. While it is fair to say that none of the reported findings come across as particularly surprising, this is an important piece of work that will help in better understanding the evolution of 5MeC. This work will also provide a useful genomic resource that will enable further evolutionary studies in the 5MeC field. My comments for improvement can be found below.

1) The authors state that the sequence data is available via SRA. However, the PRJNA589724 accession number does not work. I could also not find any reviewer links that would point to these data. This needs to be fixed before publication.
>>The data is now freely accessible.
2) The samples are poorly described. It is not clear what type of tissue / organ / cell type / developmental stage was used for DNA extraction / library preps.
**>>We have now included this as a supplemental table (Supplemental Table 1)**
3) P6, L24-25 and P7, L6-7: Targeting of TEs by 5MeC was previously shown in S maritima. A citation is required here (de Mendoza et al, 2019, Genome Res).
**>>We apologise for missing this reference in this context- we had cited it elsewhere but inadvertently omitted it here. This has now been corrected.**
4) Figure 3C. Could the authors speculate on the differences in 5MeC exon enrichment directionality? For example Limulus, Exodes, Parasteatoda show more enrichment at 5' ends whereas Bombus and Apis, display more enrichment at 3' gene ends.
**>>We cannot explain this, but do now draw the reader’s attention to these differences:**

“Among species with exon methylation, there were differences in how methylation levels changed across the gene (Figure 3C). For example, methylation was largely confined to the first three exons of *P. citri* and *N. vespilloides,* while methylation in *B. germanica* is largely found from exon four onwards (Figure 3C). However, there were no clear phylogenetic trends within these patterns suggesting patterns of methylation across genes likely change frequently during evolution.”

5) Would some of the published RNA-seq data allow for analyses of the frequency of cryptic transcription initiation in organisms with low gene body 5MeC levels vs the ones with robustly methylated GBs?
**>>We thank the reviewer for this suggestion- however we only have CAGE data, which is required for this analysis as it is the only way to robustly detect transcription initiation, from Drosophila, which has no methylation, so we don’t have anything to compare to with higher levels of methylation.**

6) Related to Figs, 4,5,6: It is not clear from which tissues / organs / cell types / developmental stages RNA-seq data originates from and how this compares to the DNA methylome data. This needs to be clearly explained. Could these discrepancies be the reason for the reversed pattern in P citri or the observation that many highly conserved and expressed genes lack 5MeC?
**>>We have included this information in the supplemental table 1.**
