# Supplementary material for: ­­­­­­Widespread conservation and lineage-specific diversification of genome-wide DNA methylation patterns across arthropods
Source: PLoS Genet. 2020 Jun 25;16(6):e1008864. doi: 10.1371/journal.pgen.1008864 (PMC7343188; doi:10.1371/journal.pgen.1008864)
Supplement: Attachment — Submitted filename: reviwsround2.docx [file pgen.1008864.s012.docx]

In addition we ask that you:
1) Provide a detailed list of your responses to the review comments and a description of the changes you have made in the manuscript.
2) Upload a Striking Image with a corresponding caption to accompany your manuscript if one is available (either a new image or an existing one from within your manuscript). If this image is judged to be suitable, it may be featured on our website. Images should ideally be high resolution, eye-catching, single panel square images. For examples, please browse our [archive](http://www.plosgenetics.org/article/browse/volume). If your image is from someone other than yourself, please ensure that the artist has read and agreed to the terms and conditions of the Creative Commons Attribution License. Note: we cannot publish copyrighted images.

We hope to receive your revised manuscript within the next 30 days. If you anticipate any delay in its return, we would ask you to let us know the expected resubmission date by email to plosgenetics@plos.org.

If present, accompanying reviewer attachments should be included with this email; please notify the journal office if any appear to be missing. They will also be available for download from the link below. You can use this link to log into the system when you are ready to submit a revised version, having first consulted our [Submission Checklist](http://journals.plos.org/plosgenetics/s/submit-now#loc-submission-checklist).

While revising your submission, please upload your figure files to the [Preflight Analysis and Conversion Engine](http://pace.apexcovantage.com/) (PACE) digital diagnostic tool. PACE helps ensure that figures meet PLOS requirements. To use PACE, you must first register as a user. Then, login and navigate to the UPLOAD tab, where you will find detailed instructions on how to use the tool. If you encounter any issues or have any questions when using PACE, please email us at figures@plos.org.

Please be aware that our [data availability policy](http://journals.plos.org/plosgenetics/s/data-availability) requires that all numerical data underlying graphs or summary statistics are included with the submission, and you will need to provide this upon resubmission if not already present. In addition, we do not permit the inclusion of phrases such as "data not shown" or "unpublished results" in manuscripts. All points should be backed up by data provided with the submission.

PLOS has incorporated [Similarity Check](http://www.crossref.org/crosscheck.html), powered by iThenticate, into its journal-wide submission system in order to screen submitted content for originality before publication. Each PLOS journal undertakes screening on a proportion of submitted articles. You will be contacted if needed following the screening process.

To resubmit, you will need to go to the link below and 'Revise Submission' in the 'Submissions Needing Revision' folder.


Please let us know if you have any questions while making these revisions.

Yours sincerely,

Wolf Reik
Section Editor: Epigenetics
PLOS Genetics


Reviewer's Responses to Questions

**Comments to the Authors:
Please note here if the review is uploaded as an attachment.**

Reviewer #1: The revisions to this manuscript are well done. This is going to make a nice contribution to the field.

Reviewer #2: I am grateful to the authors for seriously considering my concerns and revising their manuscript accordingly. The paper now far more accurately reflects the existing state of knowledge and the authors’ new contributions. I remain sceptical about the appropriateness of this manuscript for PLoS Genetics, but this is ultimately a matter for the editors.

I do have one suggestion for improvement. The conservation of gene body methylation across orthologs has been extensively studied in plants (Takuno and Gaut, PNAS 2013; Seymour et al., PLoS Genetics 2014; Takuno et al., MBE 2017; Seymour and Gaut, MBE 2019). This work reaches the same conclusion the authors do here, that the same sets of genes tend to be methylation across species (spanning diverse timescales). Furthermore, genes that retain methylation across evolution have a higher tendency to have conserved sequences and to exhibit stable and constitutive expression (a rough proxy for housekeeping functions) than genes that have species-specific methylation. This work should be cited and discussed.

>>We thank the reviewer for their positive response to our revised version of the manuscript. We have now included a section discussing the findings of gene body methylation in plants, including some discussion of the functional importance with reference to the plant *Eutrema salsugineum* in which DNA methylation at gene bodies has been lost.

I also suggest the authors test if genes that retain gbM across arthropods also have a higher tendency for conservation and stable expression, and perhaps a specific nucleosome configuration. This would make a valuable addition to the paper.

>>We thank the reviewer for this suggestion but would make the point that Figure 6 already contains analysis of the nucleosome positioning and expression variability of the genes with high levels of methylation across all arthropods, based on the mean methylation of the entire orthogroup across arthropods with gene body methylation. Within this set of genes we found clear trends for nucleosome positioning (based on orthologues in Drosophila) and gene expression variability. The set of genes that have retained gbM across arthropods that the reviewer suggests we look at will in fact overlap almost completely within this set so it is unclear what this further analysis would add.
